# Pyridopyrimidines and Pyridopyrimidinones as Kinase-Targeted Anticancer Agents: Medicinal Chemistry and Mechanistic Insights

**DOI:** 10.3390/molecules31172944

**Published:** 2026-08-22

**Authors:** Ankush Kumar, Rajwinder Kaur, Bhupinder Kumar, Rohit Bhatia

**Affiliations:** 1 Chitkara College of Pharmacy, Chitkara University, Rajpura 140401, Punjab, India; kumaranku906@gmail.com; 2 Department of Pharmaceutical Sciences, HNB Garhwal University, Srinagar 246174, Uttarakhand, India

**Keywords:** pyridopyrimidine, anticancer activity, kinase inhibitors, SAR, molecular docking

## Abstract

Pyridopyrimidine is an important heterocyclic scaffold widely explored in anticancer drug discovery. Its structural similarity to purine enables effective interaction with various biological targets, mainly kinases involved in cancer progression. This manuscript presents recent developments reported between 2021 and 2026, focusing on the biological evaluation, and structure–activity relationships of pyridopyrimidine derivatives. Many of these synthesized compounds act as inhibitors of key targets such as EGFR, CDK4/6, and the PI3K/mTOR pathway, which are closely associated with tumor growth, survival, and resistance mechanisms. Other targets such as ATR and PIM are also explored. Recent studies show that structural modifications, including substitution on the core ring and hybridization with pharmacologically active moieties like triazoles and thiazolidinediones, significantly improve anticancer activity. Several derivatives have demonstrated strong antiproliferative effects against different cancer cell lines and are capable of inducing apoptosis and cell cycle arrest. In addition, molecular docking and other computational studies support their binding efficiency and help explain their mechanisms of action. There is also increasing interest in the development of dual-target or multi-target inhibitors to overcome drug resistance and enhance therapeutic effectiveness. Overall, pyridopyrimidine- and pyridopyrimidinones-based compounds continue to show great promise as potential anticancer agents. Further research combining synthetic chemistry, biological studies, and computational approaches may lead to the development of more effective and safer drugs in the future.

## 1. Introduction

**Figure 1:**
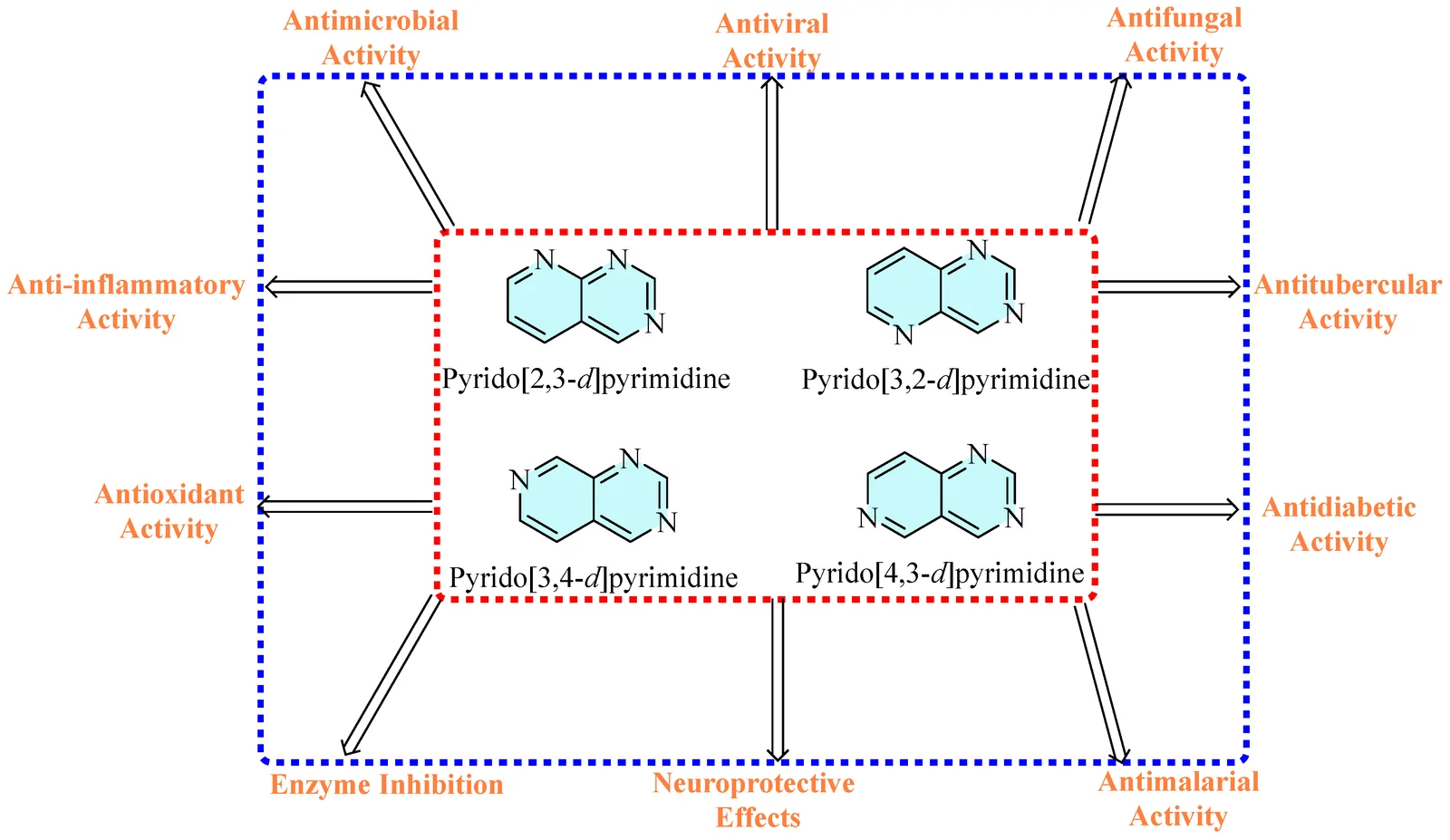
Pyridopyrimidine isomers and their associated biological activities.

Protein kinases (PKs) are a large and diverse family of enzymes that regulate numerous cellular signaling processes through the phosphorylation of specific substrates [1,2]. PKs control essential biological and physiological processes such as cell proliferation, metabolism, differentiation, and apoptosis [3]. They are also critical for maintaining cellular homeostasis [4]. Dysregulation of kinase activity by mutations, aberrant signaling and overexpression is a key driver of cancer and other proliferative disorders [5]. This makes kinases some of the most important therapeutic targets in modern drug discovery. Kinase-mediated signaling occurs through interconnected downstream pathways, including the PI3K/AKT/mTOR pathway [6], MAPK/ERK cascade [7], and cell cycle regulatory networks [8]. Key kinases such as CDKs, EGFR, PI3K, ATR, and PIM play central roles in various cellular processes [8,9,10,11,12]. Targeting the ATP-binding pocket of kinases has proven to be an effective strategy for inhibitor design [13]. Most small-molecule inhibitors act by mimicking the adenine moiety of ATP and forming key hydrogen-bond interactions with the hinge region of kinases [14,15,16]. In this context, heterocyclic scaffolds capable of reproducing these interactions have gained considerable attention. Among these, pyridopyrimidine has emerged as a privileged scaffold in medicinal chemistry [17]. Structurally, it is a fused pyridine and pyrimidine ring system and is a bioisostere of purine. This similarity allows pyridopyrimidine derivatives to be effective ATP-binding site inhibitors and form significant interactions with the hinge region [18]. The presence of more than one nitrogen atom on the ring system further increases the hydrogen-bonding ability and binding [19]. The pyridopyrimidine scaffold occurs in four isomeric forms (as displayed in Figure 1), namely, pyrido[2,3-*d*]pyrimidine, pyrido[3,2-*d*]pyrimidine, pyrido[3,4−*d*]pyrimidine and pyrido [4,3 *d*]pyrimidine. These isomers displayed diverse pharmacological properties such as antimicrobial [20], antiviral [21], antifungal, antituberculosis, antidiabetic, antimalarial, neuroprotective, anti-inflammatory, antioxidant [20] and anticancer activities [22,23]. Notably, this framework allows extensive chemical modification at several locations. Substitutions at specific positions can affect electronic, steric, and lipophilic properties, which can further affect biological activity, selectivity, and pharmacokinetic behavior.

**Figure 2:**
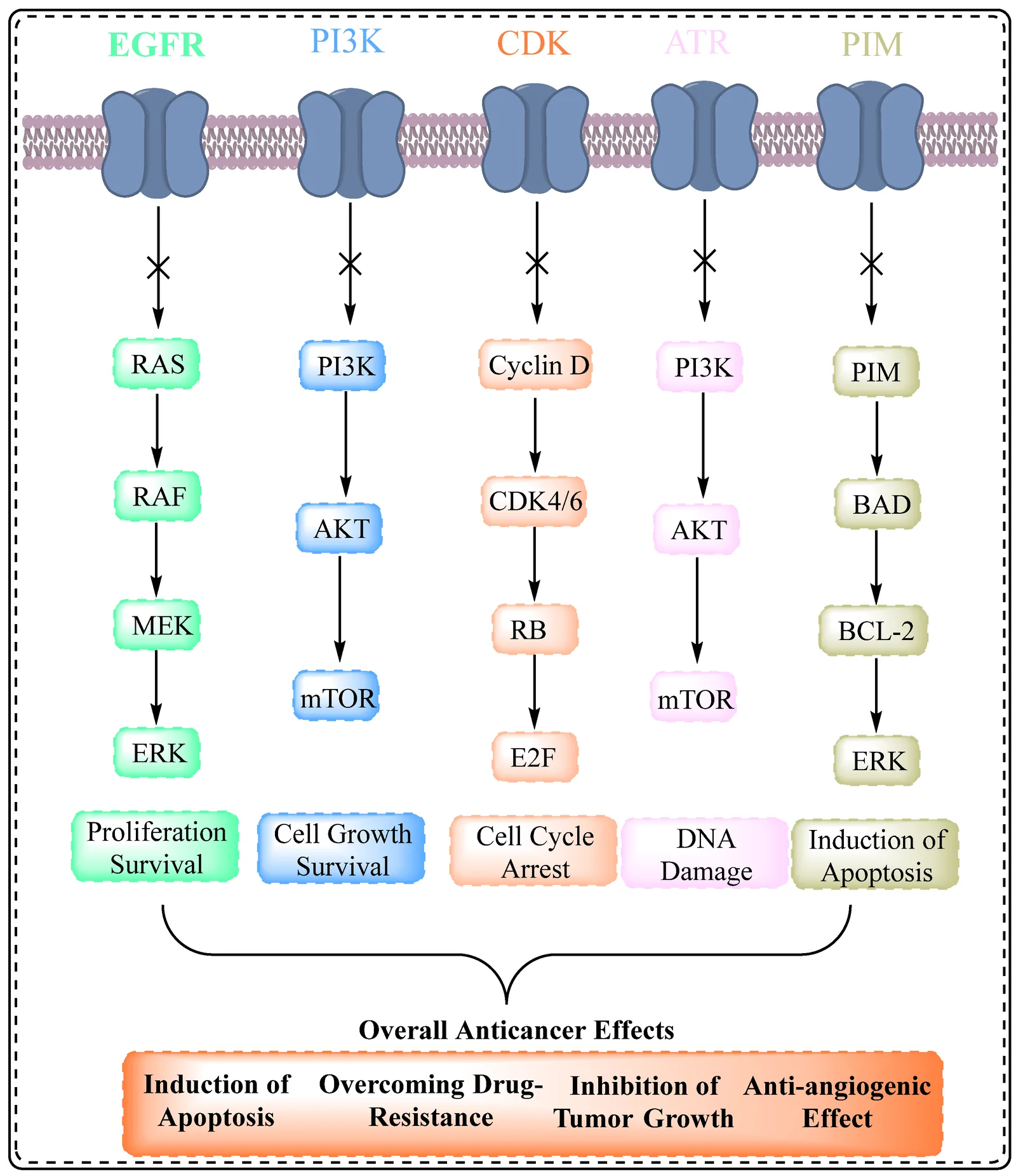
Multi-target anticancer pathway inhibition by pyridopyrimidine derivatives.

Pyridopyrimidine derivatives have emerged as promising anticancer agents due to their ability to inhibit multiple signaling pathways [24,25]. Their biological activity is associated with the inhibition of regulatory enzymes that are crucial for various cellular processes, the dysregulation of which is linked to cancer. Hence, pyridopyrimidine derivatives have exhibited potent inhibitory activity against various other kinases such as CDKs, EGFR, PI3K, ATR and PIM [8,9,10,11,12]. SAR studies have shown that the kinase inhibitory activity of the pyridopyrimidine core is greatly improved by strategic functionalization [26]. These changes allow for binding of the hinge region, as well as to neighboring hydrophobic pockets and solvent-exposed regions of the kinase active site [27]. The crosstalk among these pathways is illustrated in Figure 2. In this context, the present review summarizes the recent progress in the development of pyridopyrimidine-based kinase inhibitors, highlighting their structural diversity, SAR profiles and biological evaluation. The focus of this work is to offer a comprehensive overview of the design principles and therapeutic relevance of the pyridopyrimidine and pyridopyrimidinone scaffold in kinase-targeted drug discovery.

In recent years, pyrimidine derivatives have attracted considerable attention in medicinal chemistry, leading to the publication of several review articles covering their chemistry and therapeutic applications [17,25]. However, research in this field has advanced rapidly, particularly in the area of kinase-targeted anticancer drug discovery. The present review focuses on studies published between 2021 and 2026, a period during which significant progress has been made in the design and biological evaluation of pyridopyrimidine-based kinase inhibitors. Rather than providing a general overview of the scaffold, this review brings together recent developments across a wide range of oncogenic kinase targets, including EGFR, PI3K, CDK4/6, PIM, ATR and others. Particular emphasis is placed on SAR, biological evaluation, molecular docking studies, in vivo investigations, clinical progress, and the challenges associated with translating promising lead compounds into therapeutic candidates. By integrating these aspects, the review provides a current perspective on emerging design strategies and future opportunities for pyridopyrimidine-based anticancer agents. The literature included in this review was collected through searches of PubMed, Scopus, Web of Science and Google Scholar. The search covered publications from January 2021 to June 2026 using combinations of keywords such as pyridopyrimidine, kinase inhibitor, anticancer, EGFR, PI3K, CDK4/6, PIM, ATR, SAR, molecular docking, and cancer. Original research articles describing the design, synthesis, biological evaluation, computational studies, in vivo investigations, or clinical development of pyridopyrimidine-based kinase inhibitors were considered for inclusion. Review articles, conference abstracts, patents without experimental biological data, and studies not related to kinase-targeted anticancer therapy were excluded to maintain the focus of this review.

## 2. Pyridopyrimidines as Adenine Bio-Isosteres

Pyridopyrimidines are recognized as adenine bioisosteres due to their structural and electronic similarity to the purine nucleus of ATP [28]. The adenine moiety plays an important role in kinase–ATP interactions through hydrogen bonding with the hinge region. Pyridopyrimidine scaffolds mimic this interaction by presenting a similar arrangement of nitrogen atoms capable of forming key hydrogen bond donor–acceptor interactions, enabling competitive inhibition at the ATP-binding site [29]. The fused bicyclic structure of pyridopyrimidines closely resembles the planar geometry of adenine, which is essential for proper alignment within the kinase active site. This structural similarity allows these compounds to occupy the adenine-binding pocket and establish critical interactions with backbone residues in the hinge region. Crystallographic studies of kinase inhibitors have confirmed that heterocyclic scaffolds such as pyridopyrimidines form hydrogen bonds analogous to those of ATP, validating their role as adenine mimetics (PDB ID: 4MQ1) [30]. In addition to structural mimicry, the electronic properties of pyridopyrimidines further support their function as bioisosteres. The presence of multiple nitrogen atoms enhances their ability to participate in polar interactions and stabilize binding within the active site. These features contribute to high binding affinity and selectivity toward kinases. Clinically approved kinase inhibitors such as Palbociclib, utilize this scaffold to achieve potent ATP-competitive inhibition of CDK4/6 [31]. Their success highlights the effectiveness of pyridopyrimidines as adenine surrogates in drug design. Furthermore, structure–activity relationship studies demonstrate that substitutions around the core can modulate binding interactions, allowing the optimization of potency and selectivity. Overall, the ability of pyridopyrimidines to replicate key structural and electronic features of adenine makes them valuable scaffolds in kinase inhibitor design, with continued potential for therapeutic development.

## 3. FDA-Approved Drugs Containing Pyridopyrimidine Scaffold and Binding Modes in Kinase ATP Pocket

Early studies identified the pyridopyrimidine scaffold as a promising framework due to its ability to form key hydrogen-bond interactions with the kinase hinge region [18,32]. This led to extensive structural optimization focused on improving potency, selectivity, and pharmacokinetic properties. Palbociclib is a key example of a pyridopyrimidine-based drug that progressed from rational design to FDA approval [33]. It is a selective inhibitor of CDK4/6. The pyridopyrimidine core enables effective binding to the ATP site of these kinases. This leads to the inhibition of retinoblastoma (Rb) protein phosphorylation and arrest of the cell cycle at the G1–S phase. Preclinical studies showed strong antiproliferative activity. The effect was especially prominent in hormone receptor-positive (HR+) breast cancer models. Clinical development was carried out through the PALOMA trial program. The Phase I/II study A5481003 (PALOMA-1; NCT00721409) established the dosing schedule of 125 mg (21 days on/7 days off). It also identified neutropenia as the main dose-limiting toxicity. The Phase II results showed a significant improvement in progression-free survival (20.2 vs. 10.2 months) with Palbociclib plus letrozole [34,35]. The Phase III PALOMA-2 trial (NCT01740427) confirmed these findings. It reported a median progression-free survival of 24.8 months compared to 14.5 months with letrozole alone [36]. The Phase III PALOMA-3 trial (NCT01942135) evaluated Palbociclib with fulvestrant. It showed improved progression-free survival (9.5 vs. 4.6 months) in patients with prior endocrine therapy failure [37]. The PALOMA-4 trial (NCT02297438) further supported its efficacy and safety in broader populations. Based on these results, Palbociclib received FDA approval in 2015 [38]. It was approved for ER-positive, HER2-negative advanced or metastatic breast cancer in combination with endocrine therapy. This marked the first approval of a CDK4/6 inhibitor and highlighted the importance of the pyridopyrimidine scaffold in anticancer drug discovery. Trametinib is another FDA-approved drug as MEK1/2 inhibitors that also contains a pyridopyrimidine scaffold [39,40]. It showed significant clinical efficacy in patients with metastatic melanoma and showed a median progression-free survival of 4.8 in the phase III trial (NCT01245062) [41]. Combination with dabrafenib improved clinical outcomes. PD166326 is a pyrido[2,3−*d*]pyrimidine-containing BCR-ABL inhibitor developed as an anticancer agent [42]. It showed ABL kinase inhibitory activity with an IC_50_ of 8 nM and inhibited proliferation of K562 cells with an IC_50_ of 300 pM [42]. Pamapimod is a pyrido[2,3−*d*]pyrimidine-based p38α MAP kinase inhibitor. It was investigated as monotherapy and in combination with methotrexate (NCT00303563, NCT00316771) [43]. Piritrexim is a clinically investigated DHFR inhibitor [44]. It underwent phase I and phase II clinical studies in patients with advanced malignancies. Antitumor activity was observed in a phase I study of 51 patients with advanced cancer, while myelosuppression was observed as the dose-limiting toxicity. A phase II study was carried out in patients with metastatic urothelial cancer. Among 29 patients, 1 complete and 10 partial responses were observed, corresponding to an ORR of 38% [45]. Another phase II study reported a 27% ORR in 9 out of 33 patients, including 3 complete and 6 partial responses [46]. Collectively, these studies demonstrate that the pyridopyrimidine scaffold has emerged as a clinically relevant pharmacophore.

Table 1 describes the various characteristics of the FDA-approved drug Palbociclib.

**Figure 3:**
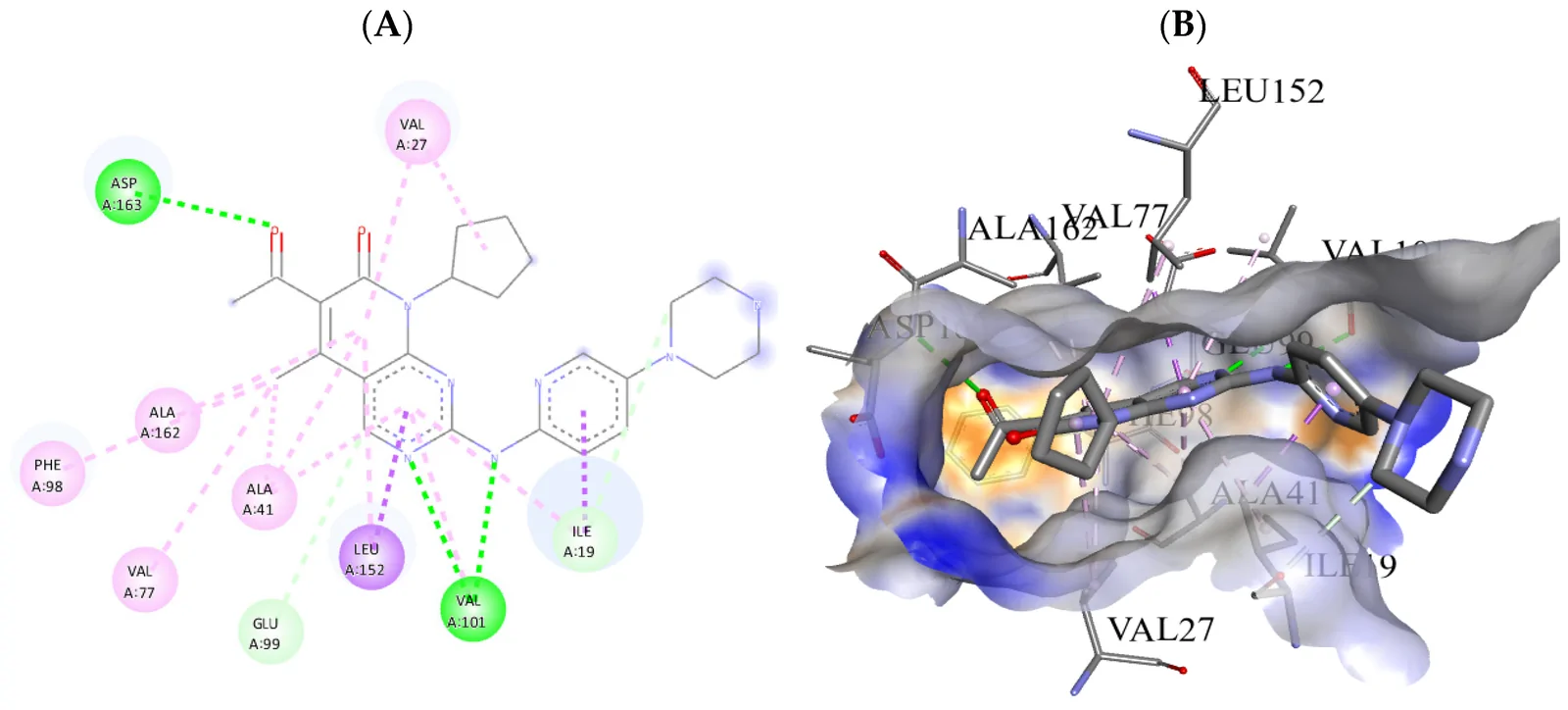
2D (**A**) and 3D (**B**) pose of Palbociclib within the CDK6 active site.

To determine the binding mode of Palbociclib, we performed molecular docking studies using AutoDock 4.2 within the CDK6 active site (PDB ID: 5L2I) [47]. The protein structure was prepared by removing the co-crystallized ligand and water molecules, followed by the addition of polar hydrogen atoms. Kollman charges were also added. For the preparation of the ligand, the chemical structure of Palbociclib was converted into the PDBQT format. A grid box centered at x = 14.767, y = 28.828 and z = 5.797 with dimensions of 40 × 42 × 40 Å was defined. To validate the docking protocol, the co-crystallized ligand was extracted from the CDK6 crystal structure and redocked into the ATP-binding site using the same parameters. The redocked pose closely reproduced the binding orientation with an RMSD of 1.674 Å. This confirms the reliability of the docking protocol for subsequent docking studies. The docked pose (displayed in Figure 3) of Palbociclib showed a stable conformation in the ATP-binding pocket, forming key hydrogen-bond interactions with the hinge region residue Asp163. The cyclopentyl ring formed hydrophobic interactions with Val27. The pyrimidine ring and amino group formed a hydrogen-bond interaction with Val103. Additional hydrophobic interactions with residues such as Phe98, ALA41, LEU152, and ILE19 contribute to the stabilization of the ligand within the binding cavity. The binding orientation observed in the docking study is consistent with reported crystallographic data, confirming proper hinge region binding and validating the docking protocol [47,48].

## 4. Pyridopyrimidines and Pyridopyrimidinones as Potent Kinase Inhibitors

Pyridopyrimidines and pyridopyrimidinones derivatives display potent anticancer activities against various kinases. In this section, we compile the SAR and biological evaluation of pyridopyrimidine derivatives as kinase inhibitors. Structural modifications of the core scaffold significantly influence activity against key targets such as PIM, EGFR, PI3K, ATR, and CDKs. Biological studies indicate strong antiproliferative effects, and SAR studies highlight the role of substituents in improving potency and selectivity.

### 4.1. Pyridopyrimidines and Pyridopyrimidinones as PIM Inhibitors

**Figure 4:**
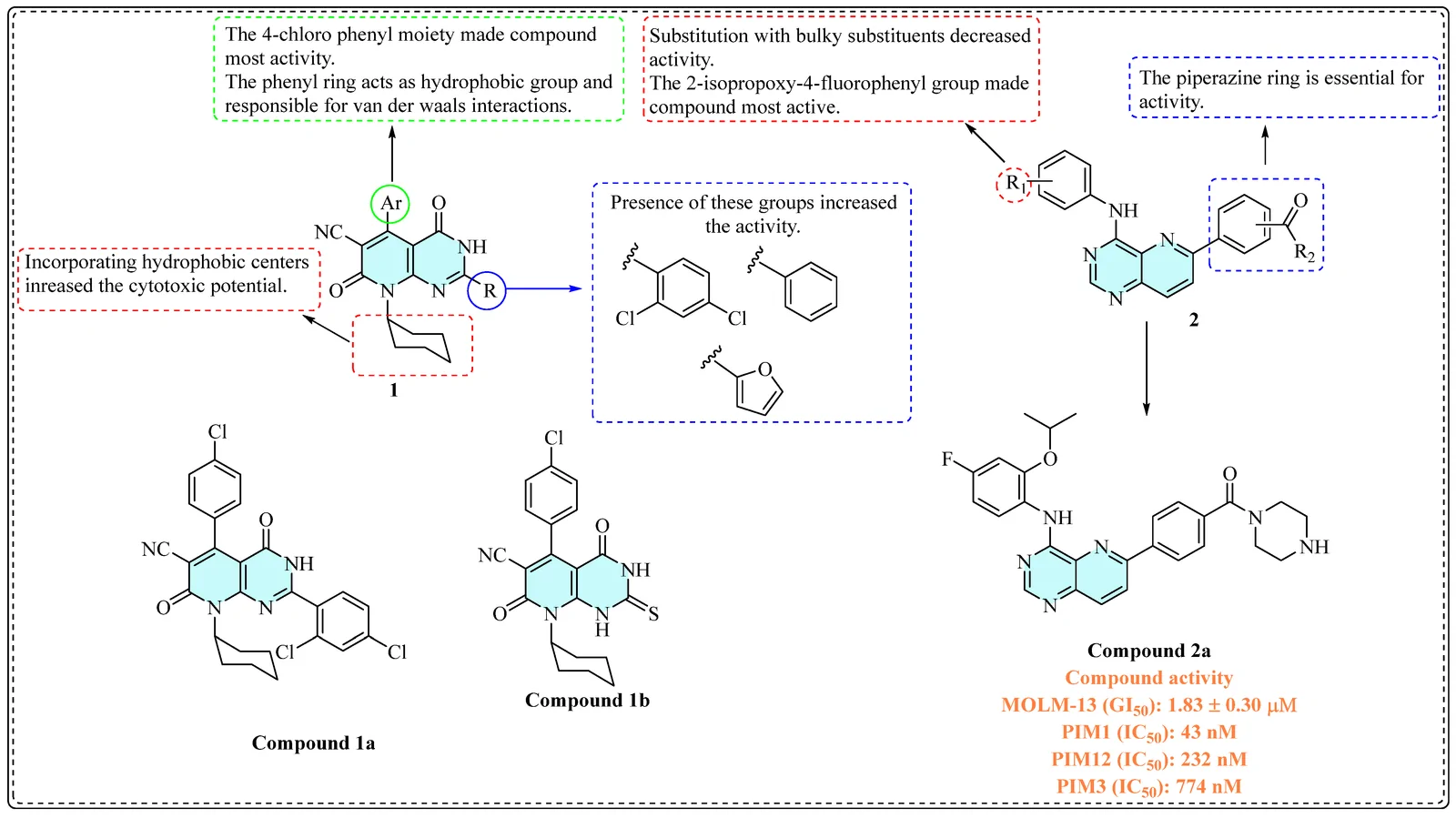
Chemical structures and SAR of PIM kinase inhibitors.

Pyrido[2,3-*d*]pyrimidine derivatives (**1**) were synthesized by Tantawy and co-workers through acylation or thioacylation of aminonicotinonitrile, followed by intramolecular heterocyclization [49]. Among all the synthesized compounds (displayed in Figure 4), two compounds, **1a** and **1b** were found to be potent PIM-1 kinase inhibitors, showing IC_50_ values of 11.4 and 17.2 nM, respectively, as compared with the reference drug staurosporin (IC_50_: 16.7 nM). Compound **1a** also exhibited excellent cytotoxicity against MCF-7 cells, with an IC_50_ of 0.57 µM, compared to staurosporin (IC_50_: 6.76 µM). Both compounds also displayed the highest inhibition of 97.8 and 94.6% as compared to staurosporin (95.6%). Additionally, the compound induced cell cycle arrest at the G1 phase of the cell cycle. SAR studies are displayed in Figure 4. It was found that the presence of 4-phenyl ring on the pyrimidine ring showed excellent PIM-1 kinase inhibitory activity [49]. Han and co-workers optimized the 4,6-disubstituted pyrido[3,2-*d*]pyrimidines (**2**) as dual MNK/PIM inhibitors for the treatment of leukemia. Antiproliferative activity was evaluated against the MOLM-13 cell line, and compound **2a** displayed potent inhibition with a GI_50_ of 1.83 µM. Compound **2a** also exhibited potent MNK1 and MNK2 inhibitory potential with IC_50_ of 1 and 7 nM. Moreover, compound **2a** exhibited PIM1/2/3 inhibitory activity with IC_50_ of 43/232/774 nM. The binding affinity of compound **2a** was compared with SGI-1776. Compound **2a** displayed docking score of −6.434 kcal/mol and SGI-1776 showed −7.450 kcal/mol. Compound **2a** also induced cell cycle arrest in the G1 phase which showed that apoptosis induction is due to the inhibition of PIMs. The antileukemic effects of compound **2a** were tested in a MOLM-13 xenograft model of mice, and compound **2a** was given at doses of 5 and 10 mg/kg ip daily for two weeks. It inhibited growth with inhibition rates of 41.22 and 57.65%, respectively. Protein analysis was also carried out using Western blotting, and the results displayed that compound **2a** at 10 mg/kg reduced the levels of cyclin D1, *p*-BAD, *p*-eIF4E and *p*-4EBP1. But, at 5 mg/kg compound **2a** only decreased the level of *p*-4EBP1. This indicates that the in vivo inhibition of cells is associated with PIM and MNK inhibition [50]. The SAR study and chemical structures are displayed in Figure 4. Although compounds synthesized by Tantawy and Han et al. exhibited potent PIM kinase inhibition, their activity was influenced by different structural features. In the series synthesized by Tantawy et al., the 4-chlorophenyl group was important for PIM-1 inhibitory activity, while replacement with any other hydrophobic aromatic group enhanced cytotoxicity. In another series designed by Han et al., compound **2a** containing a 2-isopropoxy-4-fluorophenyl substituent along with a piperazine moiety, demonstrated high potency and improved in vivo efficacy. These studies indicate that both the pyridopyrimidinone and pyridopyrimidine nuclei serve as effective ATP-mimetic scaffolds.

### 4.2. Pyridopyrimidines as PI3K Inhibitors

**Figure 5:**
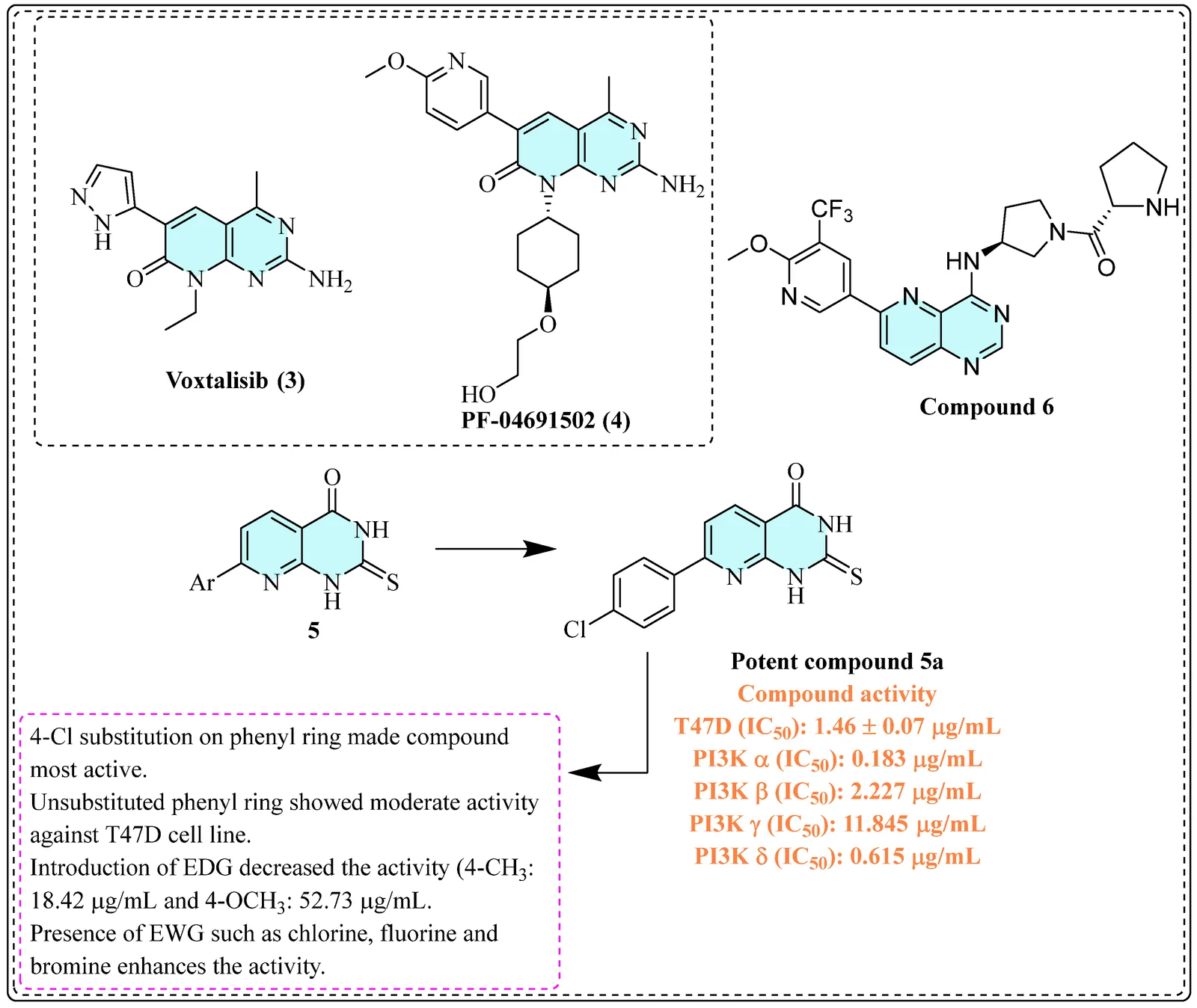
Chemical structures and SAR of pyridopyrimidines as PI3K inhibitors.

Voxtasilib (**3**) is a dual mTOR/PI3K inhibitor, also known as XL765. It is a pyridopyrimidine derivative that was found to be effective in patients with metastatic breast cancer and showed progression-free survival of 24 weeks in 22% of patients [51,52]. It also acts as a PI3K pathway modulator and has the ability to induce apoptosis [53]. PF-04691502 (**4**) is a potent and selective PI3K and mTOR inhibitor with an IC_50_ of 32 nmol/L [54]. It also triggers apoptosis via the activation of the mitochondrial pathway and caspases 3 and 9 [55]. Considering these facts, Ali and co-workers synthesized new pyridopyrimidine derivatives (**5**) as PI3K inhibitors. All the synthesized derivatives displayed GI% in the range of 50 to 104% against several cancer cell lines [56]. Compound **5a** showed potent T47D inhibition with an IC_50_ of 1.46 µg/mL, compared to pictilisib (IC_50_: 2.56 µg/mL). In vitro results displayed that the unsubstituted phenyl ring showed moderate activity of 16.09 µg/mL. The introduction of EDGs such as methyl and methoxy reduced the activity (18.42 and 52.73 µg/mL). EWGs on the phenyl ring are essential for T47D inhibition. Compound **5a** did not show any cytotoxic effect on the MCF-10A normal cell line with an IC_50_ of 421.62 µg/mL. Compound **5a** also induced cell cycle arrest in the G2-M phase. Proapoptotic activity was also observed, and the results showed an increase in the cells, with early apoptosis rising from 0.64 to 18.83% and late-stage apoptosis from 0.13 to 11.51%. Western blotting results showed 2.38-fold upregulation of Bax and a 0.69-fold reduction in the overexpression of Bcl-2. So, this cellular death in the T47D cells is linked with the activation of intrinsic pathways. An in vitro PI3K inhibitory assay was carried out on four isoforms such as PI3K α, PI3K β, PI3K γ and PI3K δ. Results showed an IC_50_ of 0.183, 2.227, 11.845 and 0.615 µg/mL, compared to the reference drug pictilisib (IC_50_: 0.097, 0.263, 0.095 and 0.024 µg/mL against PI3K α, PI3K β, PI3K γ and PI3K δ, respectively). Docking studies were carried out on PI3Kα (PDB ID: 4JPS). Compound **5a** showed a docking score of −10.74 kcal/mol, comparable to that of the co-crystallized ligand (−8.8 kcal/mol). The nitrogen in the pyrimidine ring formed a hydrogen-bond interaction with Val851. The thioxo group also formed hydrogen bonds with Arg852 and Ser854 [56]. Novel pyrido[3,2-*d*]pyrimidine derivatives as PI3Kδ were synthesized by Bai et al., and compound **6** was found to be a potent PI3Kδ inhibitor with an IC_50_ of 2.82 nM. Antiproliferative activity was also evaluated, demonstrating an IC_50_ of 0.035 µM [57]. The chemical structures of various PI3K inhibitors are displayed in Figure 5. The above-mentioned PI3K inhibitors demonstrate that peripheral substituents strongly influence biological activity. Compound **5a** showed superior potency due to the presence of the 4-chlorophenyl group. Electron-donating substituents such as methoxy and methyl reduced activity. Compound **6** indicates that both the substituent and the scaffold are important for improving potency and isoform selectivity.

### 4.3. Pyridopyrimidines and Pyridopyrimidinones as EGFR Inhibitors

**Figure 6:**
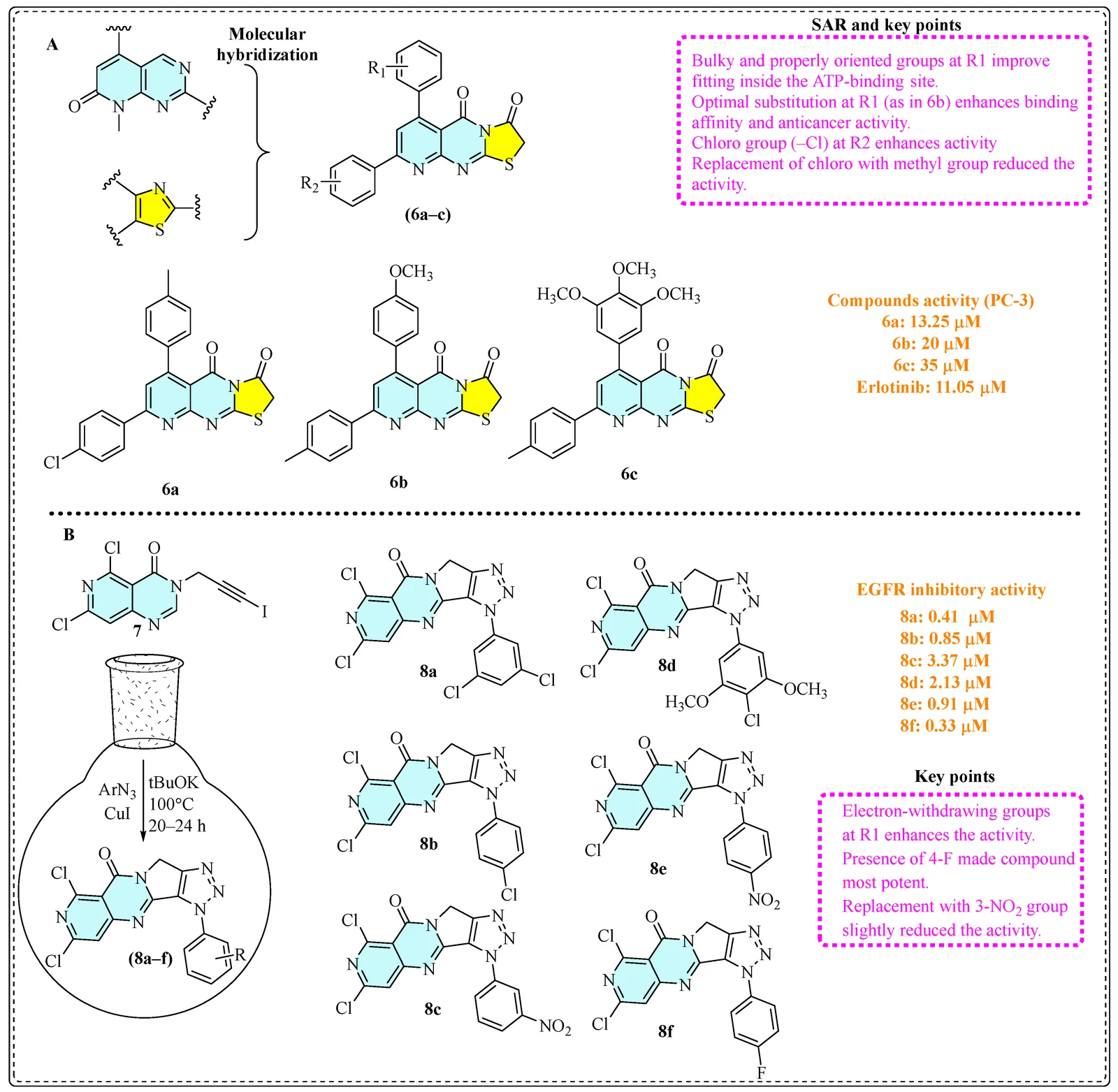
Chemical structures of EGFR inhibitors. (**A**) Chemical structures and SAR of novel pyrido-thiazolo-pyrimidinone hybrids synthesized by Alasfoury and co-workers. (**B**) Chemical structures and SAR of fused 1,2,3-triazoles containing pyrido[4,3−*d*]pyrimidinone hybrids.

Alasfoury and co-workers synthesized novel pyrido-thiazolo-pyrimidinone hybrids (**6a–c**) (displayed in Figure 6A) through a molecular hybridization technique targeting EGFR^WT^ and EGFR^T790M^ [58]. Three compounds, **6a**, **6b** and **6c**, displayed potent cytotoxic activity against the PC-3 cell line with IC_50_ values of 13.25, 20 and 35 µM. Docking studies were performed on EGFR^WT^ and EGFR^T790M^ using PDB IDs of 4HJO and 3W2O, respectively. All the compounds displayed binding free energies in the range of −1.71 to −4.79 kcal/mol against EGFR^T790M^ and −5.24 to −10.98 kal/mol against EGFR^WT^. Docking studies showed that the thiazole moiety of compound **6a** formed hydrogen bonds with Met790 and Gln791 amino acids. Additionally, the tricyclic moiety formed hydrophobic interactions with Ala743, Leu792, Pro794 and Phe792 [58]. Triazole-based compounds exhibit various biological activities, including anticancer activity [59]. Pyrido[4,3−*d*]pyrimidinone derivatives have also shown activity against various types of cancer [60]. The authors synthesized new fused 1,2,3-triazoles-containing pyrido[4,3−*d*]pyrimidinone hybrids (**8a–f**) by reacting iodoalkyne (**7**) with aryl azide in the presence of a copper catalyst and potassium *tert*-butoxide as EGFR inhibitors (displayed in Figure 6B). Among all the synthesized derivatives, six compounds, **8a**, **8b**, **8c**, **8d**, **8e** and **8f**, displayed potent EGFR inhibitory activity in the range of 0.33 to 3.37 µM as compared with the standard drug erlotinib (0.43 µM). In vitro cytotoxic activity was also evaluated against the MCF-7, NCI-H460 and A549 cell lines. Two compounds, **8a** and **8f**, showed potent activity against MCF-7 with IC_50_ values of 3.91 and 3.30 µM. SAR studies displayed that the presence of EWGs such as fluorine and 3,5-diCl groups is important for inhibitory activity. The butyl and methyl groups reduce the inhibitory activity against MCF-7 (IC_50_: 38.28 µM and 28.45 µM). Furthermore, docking studies were carried out on the EGFR protein with PDB ID of 4HJO. All the synthesized derivatives displayed binding affinities in the range of −8.94 to −9.80 kcal/mol. Compound **8e** showed the highest binding score of −9.80 kcal/mol. Compound **8f** formed h-bonds with Lys721. ADMET results showed that all the compounds followed Lipinski’s rule of five without any deviations [61].

**Figure 7:**
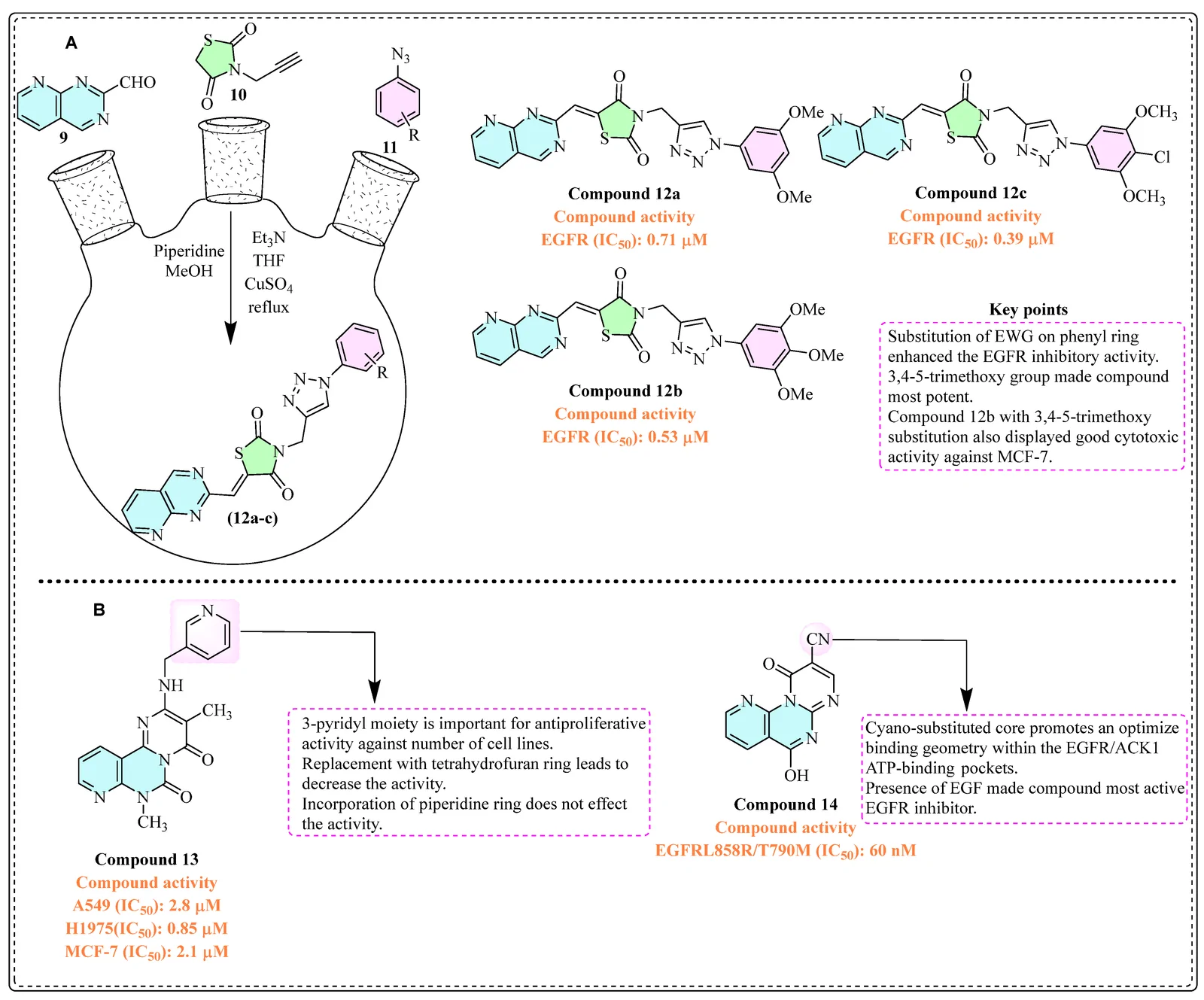
Chemical structures of EGFR inhibitors. (**A**) Designing strategy and chemical structures of pyrido[2,3−*d*]pyrimidine. (**B**) Chemical structures and SAR of fused pyridopyrimidopyrimidines.

Pyridopyrimidines are found as an emerging chemical moiety due to their diverse biological activities [17,25]. Similar to other heterocyclic rings, thiazolidine-2,4-dione has displayed various biological and chemical activities [62]. Some compounds, such as GSK1059615, AZD1208, and SMI-4a, are being investigated in clinical trials for their potential anticancer activity. [63,64]. Molecular hybridization of thiazolidine-2,4-dione with other pharmacophores has displayed many biological activities [65,66,67]. Based on these findings, three pharmacophores, thiazolidine-2,4-dione, 1,2,3-triazole and pyrido[2,3−*d*]pyrimidine, were combined in a single framework to synthesize the desired compounds. Pyrido[2,3−d]pyrimidine-2-carbaldehyde (**9**), 3-(prop-2-yn-1-yl)thiazolidine-2,4-dione (**10**) and aryl azides (**11**) were mixed in methanol, THF and CuSO_4_ and refluxed for 20 h to get the products as pale-red solids (**12a–c**). Two compounds, **12b** and **12c**, displayed potent antiproliferative activity against MCF-7 cells with IC_50_ values of 4.19 and 3.52 µM as well as EGFR inhibitory activity of 0.53 and 0.39 µM. Compound **12a** with 3,5-dimethoxy group also displayed comparable EGFR inhibitory activity of 0.71 µM. The chemical structures and SAR are displayed in Figure 7A. Docking studies were performed with the EGFR protein (PDB ID: 1M17) using erlotinib as an internal ligand. Compound **12b** showed better binding affinity of −105.89 kcal/mol as compared to erlotinib (−101.98 kcal/mol). The methoxy groups of compound **12b** formed hydrogen-bond interactions with Met742 and Lys721. Other hydrophobic interactions were also found with Val702, Ala719, Cys773 and Leu820. AMDET studies also showed that the Lipinski parameters are within range and no violation was observed [68]. El-Essawy et al., synthesized fused pyridopyrimidopyrimidines (Figure 7B) as potent dual EGFR/ACK1 inhibitors. Two compounds, **13** and **14**, showed potent anticancer activity against a panel of cells such as A549, H1975, MCF-7, HCT-116, HepG2, WI-38 and MRC-5. The two compounds displayed activity in the range of 0.42 to 10.4 µM. Compound **14** showed an excellent IC_50_ of 0.42 µM against H1975 cells. Furthermore, the two lead compounds, **13** and **14**, were evaluated against EGFR kinase. Compound **14** showed potent EGFR inhibitory activity with an IC_50_ of 60 nM. Compound **14** also showed the strongest apoptotic response of 32% (total apoptotic fraction) which was higher than that of the vehicle control (0.6%). It also induced cell cycle arrest in the G1 phase of the cell cycle. SAR studies showed that substitution at the C-7 position modulated the potency. The compound with a 3-pyridylmethylamino group increased the hydrophobic interaction but exhibited lower aqueous solubility of 28 µg/mL [69].

**Figure 8:**
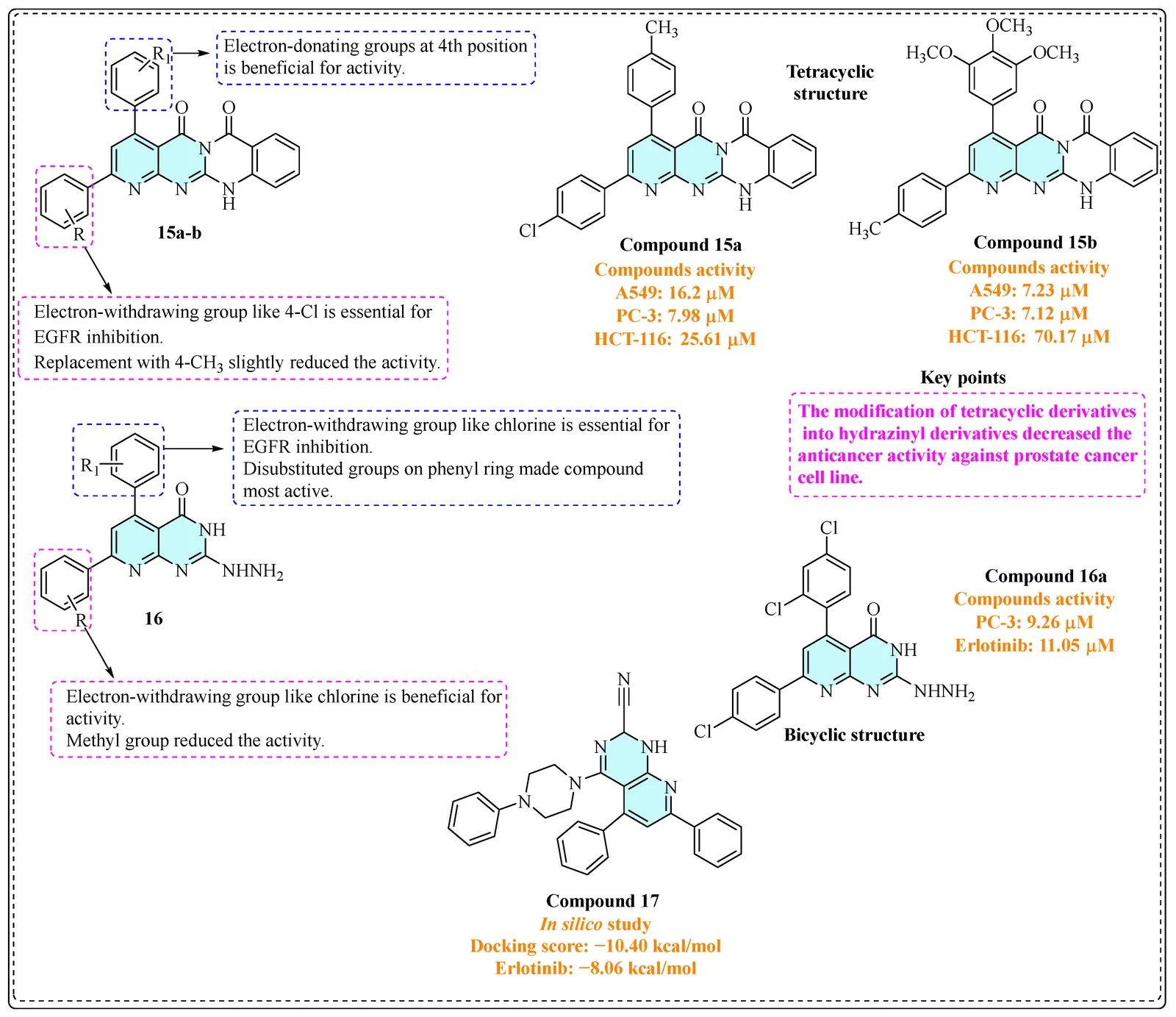
Chemical structures and SAR of EGFR inhibitor.

Elzahabi and co-workers synthesized new pyrido[2,3−*d*]pyrimidin-4(3*H*)-one derivatives (**15a-b**) as potent EGFR^WT^ and EGFR^T790M^ inhibitors and apoptotic inducers [70]. Antiproliferative activity was carried out against a number of cell lines such as the A549, PC-3 and HCT-116 cell lines. Two compounds showed good inhibition. Compound **15a** showed an IC_50_ values of 16.2, 7.98 and 25.61 µM against the A549, PC-3, and HCT-116 cell lines. Compound **15b** showed an IC_50_ of 7.23, 7.12 and 70.17 µM against the A549, PC-3, and HCT-116 cell lines as compared to erlotinib (6.53, 11.05 and 5.47 µM against A549, PC-3, and HCT-116 cell lines). These compounds were further evaluated for EGFR kinase inhibitory activity. Compounds **15a** and **15b** showed IC_50_ values of 0.099 and 0.419 µM against EGFR^WT^ as compared to erlotinib (0.043 µM). **15a** and **15b** also showed an IC_50_ of 0.123 and 0.290 µM against EGFR^T790M^ as compared to erlotinib (0.071 µM). Cell cycle analysis showed that compound **15a** causes a cell cycle increase at the pre-G1 and S phases by 22- and 1.3-folds, respectively. The cell cycle phase was increased from 41.03% to 53.69% in Pc-3 cells. Compound **15a** also increased the early apoptosis ratio from 0.43 to 13.92%. It also showed an increase in late apoptosis from 0.15 to 22.49% (150-fold). All these results showed that compound **15a** has an apoptotic effect on PC-3 cells. SAR studies showed that electron-donating groups at the fourth position are important for anticancer activity. Compound **15a** showed binding free energies of −19.29 and −15.63 kcal/mol against EGFR^WT^ and EGFR^T790M^. Compound **15b** showed binding free energies of −21.92 and −19.30 kcal/mol against EGFR^WT^ and EGFR^T790M^ [70]. They have also synthesized another series of two heteroaromatic rings (**16**). Compound **16a** was found as a potent anticancer agent with an IC_50_ of 9.26 µM compared to erlotinib (11.05 µM). Compound **16a** also showed potent EGFR^WT^ and EGFR^T790M^ inhibitory activities of 0.594 and 0.571 µM, respectively. It also displayed docking scores of −15.80 and −12.15 kcal/mol against EGFR^WT^ and EGFR^T790M^. Garmabi et al. synthesized pyrido[2,3−*d*]pyrimidine derivatives and evaluated them for EGFR activity through a docking technique [71]. Docking studies showed a binding score in the range of −9.01 to −10.40 kcal/mol. Among them, compound **17** showed a high docking score of −10.40 kcal/mol as compared to the drug erlotinib (−8.06 kcal/mol). Compound **17** formed hydrophobic interactions with Met769 and Cys773. Furthermore, ADMET results showed molecular weights t from 377.44 to 468.55 g/mol and TPSA between 65.70 and 74.93 Å. Log *p* values ranged from 3.53 to 4.71. All compounds also showed good synthetic accessibility (from 2.97 to 3.52) [71]. Figure 8 showed the chemical structures and SAR of EGFR inhibitors.

**Figure 9:**
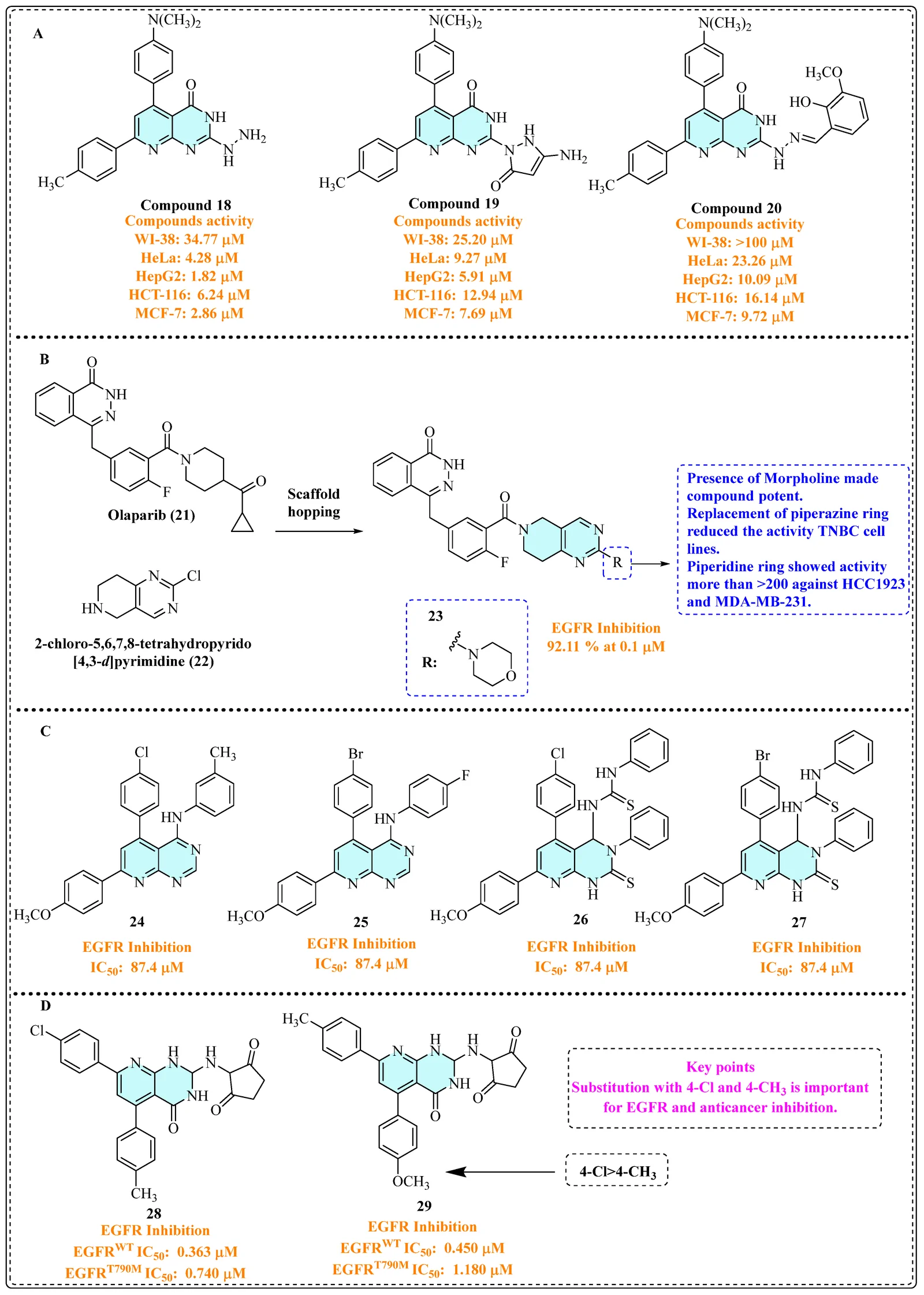
Chemical structures and SAR of EGFR inhibitor. (**A**) Chemical structures of new pyridopyrimidine derivatives, (**B**) Designing strategy of novel pyrido[4,3−*d*]pyrimidine derivatives by scaffold hopping technique and SAR. (**C**) Chemical structures of pyridopyrimidine derivatives synthesized by Eissa and co-workers. (**D**) Chemical structures and SAR of pyrido[2,3−*d*]pyrimidin-4(3*H*)-one derivatives.

Krakisha et al., synthesized new pyridopyrimidine derivatives (Figure 9A) as dual EGFR and CDK4 inhibitors and as potent anticancer agents [72]. Three compounds, **18**, **19** and **20**, displayed potent antiproliferative activity against the WI-38, HeLa, HepG2, HCT-116 and MCF-7 cell lines. Compound **18** displayed IC_50_ values of 34.77, 4.28, 1.82, 6.24 and 2.86 µM. Compound **19** displayed IC_50_ values of 25.20, 9.27, 5.91, 12.94 and 7.69 µM. Similarly, compound **20** displayed IC_50_ values of >100, 23.26, 10.09, 16.14 and 9.72 µM compared to doxorubicin (6.72, 5.57, 4.50, 5.23 and 4.17 µM). All three compounds, **18** (IC_50_: 96 ng/mL), **19** (IC_50_: 3.114 µM against HepG2 and 2.852 µM against MCF-7) and **20** (IC_50_: 4.479 against HepG2 and 3.382 against MCF-7) were compared to erlotinib (IC_50_: 2.247 µM against HepG2). SAR studies revealed that compound **19**, in which a pyridopyrimidine ring is fused with pyrazole ring, exhibited the greatest potency. The fused pyridopyrimidine with an unsubstituted triazole ring displayed moderate activity. The presence of a substituted triazole with amino or aryl groups exhibited weak activity. Also, two distinct EDGs, such as hydroxy and methoxy groups, in compound **20** improved the activity. Also, the presence of EWGs such as 3-NO_2_, 4-Br and 4-Cl reduced the activity [72]. Lin and co-workers synthesized novel pyrido[4,3−*d*]pyrimidine derivatives (as displayed in Figure 9B) by scaffold hopping technique (Olaparib (**21**) + 2-chloro-5,6,7,8-tetrahydropyrido[4,3−*d*]pyrimidine (**22**)) as dual PARP and EGFR inhibitors. Compound **23** was found to be a potent antiproliferative compound with IC_50_ values of 3.23, 15.6, >200, 199.2, 18.7 and 50 µM against the HCC1937, MDA-MB-468, MDA-MB-231, MCF-7, MX-1 and BRCA1/2. Compound **23** also displayed a potent EGFR inhibition rate of 92.11% at 0.1 µM. Compound **23** also reduced the phosphorylated EGFR level [73]. Eissa and co-workers synthesized pyridopyrimidine derivatives (Figure 9C) as potent EGFR inhibitors [74]. Four compounds, **24**, **25**, **26** and **27**, displayed potent anticancer activity against the MCF-7 and MDA-MB-231 cell lines. Compounds **24**, **25**, **26** and **27** showed IC_50_ values of 4.82, 12.70, 32.70 and 10.80 µM against MCF-7 and 4.43, 11.90, 29.80 and 22.20 µM against MDA-MB-231. Furthermore, all these four compounds showed EGFR inhibitory activity of 87.04, 38.73, 35.44 and 31.24 µM [75]. Nossier and co-workers synthesized pyrido[2,3−*d*]pyrimidin-4(3*H*)-one derivatives as potent EGFR^WT^ and EGFR^T790M^ inhibitors (Figure 9D). Two compounds, **28** and **29**, displayed potent inhibitory activity of 14.01 and 18.45 µM against the PC-3 cell line compared to the reference drug erlotinib 11.05 µM. SAR studies displayed that the 4-OCH_3_ group displayed superior activity to 4-CH_3_. Further, an EGFR enzymatic assay was carried out on EGFR^WT^ and EGFR^T790M^. Compound **28** showed IC_50_ values of 0.363 and 0.740 µM against EGFR^WT^, while compound **29** showed 0.450 and 1.180 µM EGFR^WT^, compared to the reference drug erlotinib (IC_50_: 0.051 and 0.094 µM) [74].

The reported EGFR-targeting pyridopyrimidine derivatives showed good selectivity and potency against EGFR^wt^ and EGFR^T790^. The incorporation of electron-withdrawing groups such as fluoro, chloro and 3,5-dichloro enhanced the EGFR inhibitory activity. However, electron-donating groups, such as the 4-methoxy group in compound **28** and the 3,5-dimethoxy derivative in **12a**, displayed promising EGFR inhibitory activity. SAR studies demonstrated that the 4-OCH3 group displayed superior activity to 4-CH_3_. Compound **14** showed an EGFR^L858R/T790M^ inhibitory activity of 60 nM. A cyano-substituted core promotes binding efficacy within the EGFR ATP-binding pocket. These findings suggest that biological inhibition not only depends on the main core scaffold, but the electronic nature of the substituents is also important for proper orientation within the EGFR active site.

### 4.4. Pyridopyrimidines Derivatives as CDK Inhibitors

**Figure 10:**
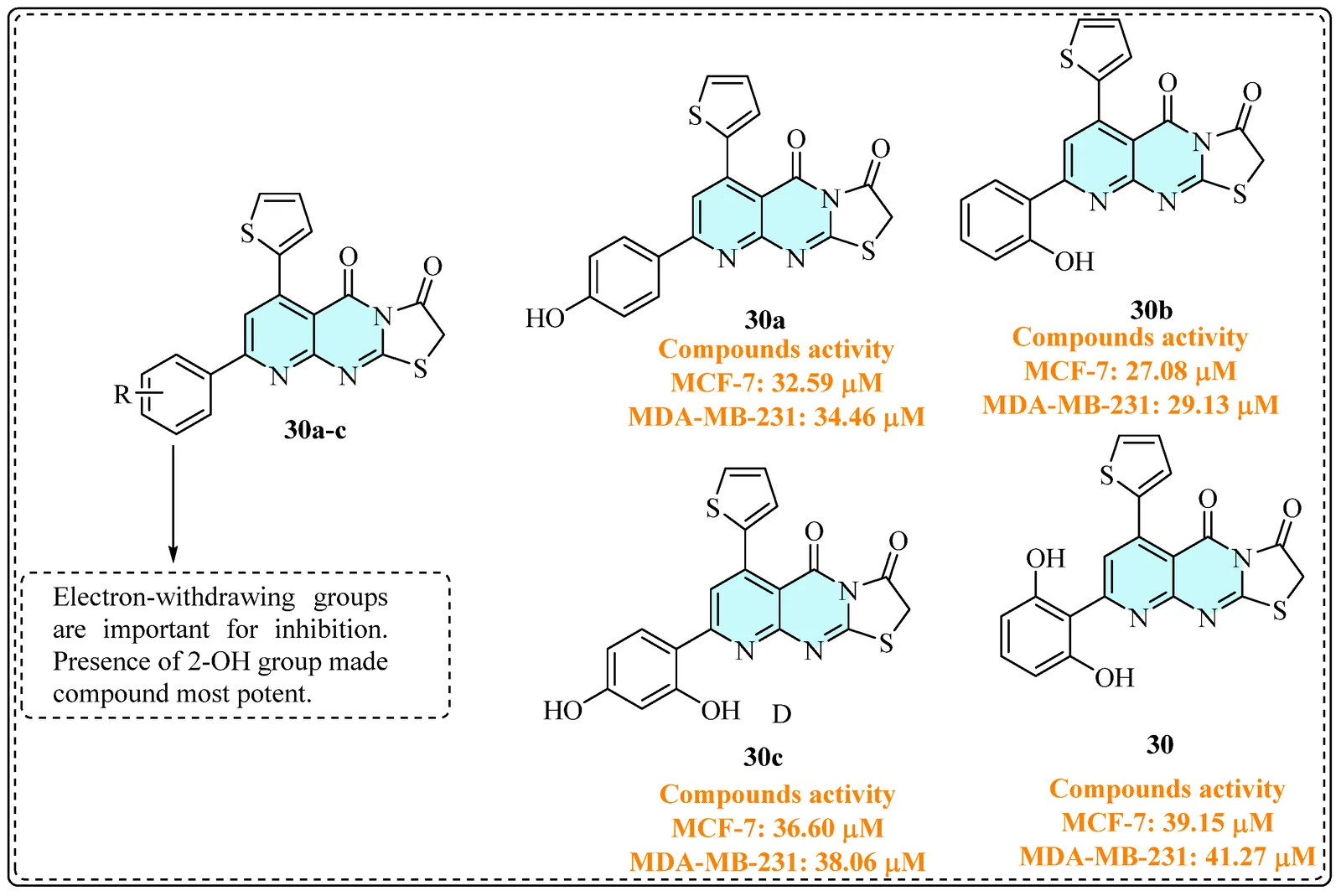
Chemical structures and SAR of Thiazolo-pyridopyrimidines (**30a–d**).

Thiazolo-pyridopyrimidines (**30a–d**) were synthesized by Shetty et al. as CDK4/6 inhibitors, and their anticancer activity was evaluated against breast cancer cell lines (MCF-7 and MBA-MD-231). In silico screening was carried out to find the hit molecules. In silico docking studies were carried out on CDK4 and CDK6 with the PDB IDs of 2W96 and 6OQO. Docking studies with CDK4 protein showed that the nitrogen atom of the pyrimidine ring formed hydrogen bonds with Lys35. The ketone group at the C3 position of the thiazole ring also formed a hydrogen-bond interaction with Val96 and the 4-OH group formed an interaction with Lys142. The ketone group of the thiophene ring formed a hydrogen bond with Lys35. Four compounds, **30a**, **30b**, **30c** and **30d**, showed docking scores of −7.11, −6.461, −6.247 and −6.383 kcal/mol against CDK4 and −6.765, −7.137, −6.747 and −6.838 kcal/mol against CDK6. RMSD values were found in the range of 1 to 3 Å. In vitro anticancer results showed that compound **30b** displayed potent activity with IC_50_ values of 27.08 and 29.13 µM against MCF-7 and MDA-MB-231 [76]. Figure 10 displays the chemical structures of thiazolo-pyridopyrimidines.

### 4.5. Pyridopyrimidine as ATR Inhibitors

**Figure 11:**
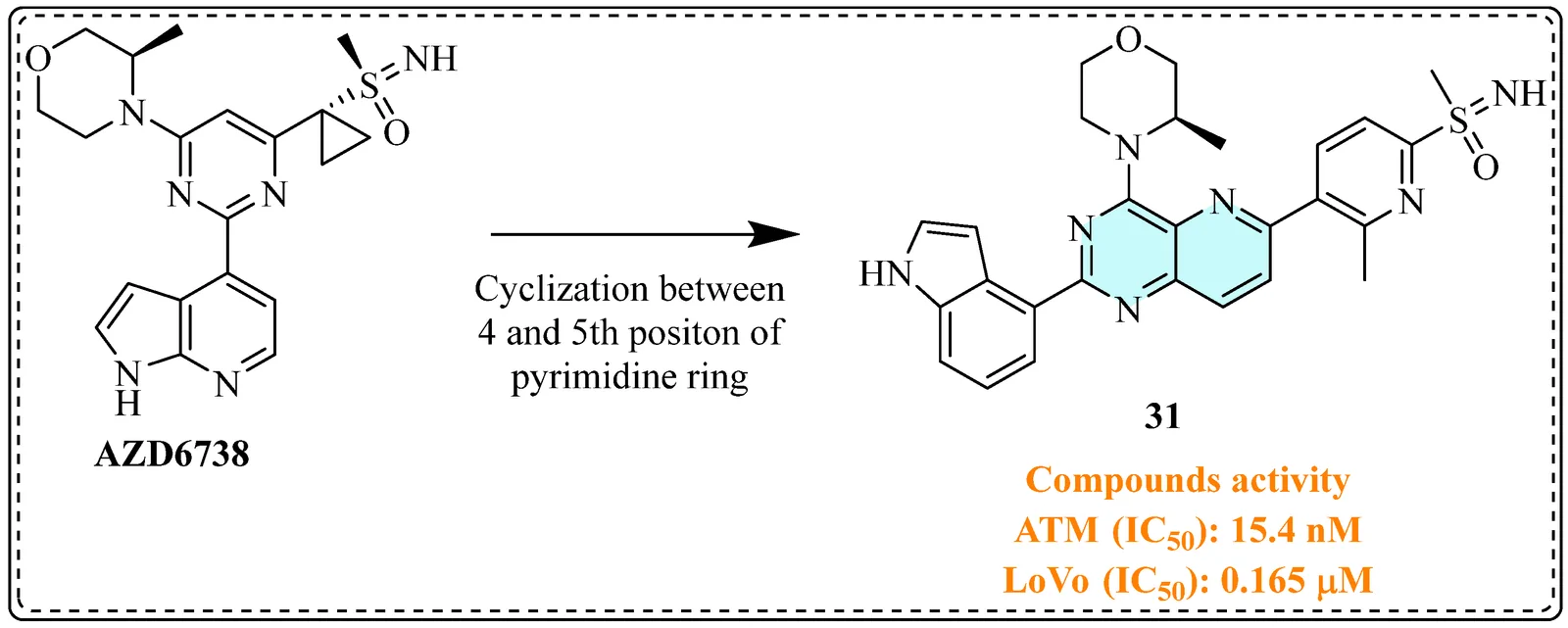
Chemical structures and SAR of pyrido[3,2−*d*]pyrimidine derivatives as novel ATR inhibitors.

Duan et al. synthesized pyrido[3,2−*d*]pyrimidine derivatives as novel ATR inhibitors. Compound **31** displayed potent ATR inhibitory activity of 15.4 nM. Antiproliferative activity was evaluated against the ATM-deficient LoVo cell line, and compound **31** showed an IC_50_ of 0.165 µM compared to AZD6738. Furthermore, a docking study was carried out on 5UK8, in which the 3-methylmorpholine group formed a hydrogen bond with Val851. The NH of the indole moiety formed hydrogen bond with Asp810. Compound **31** also induced cell cycle arrest in the S and G2/M phase [77]. Figure 11 displays the chemical structures of pyrido[3,2−*d*]pyrimidine derivatives as novel ATR inhibitors. The pyridopyrimidine scaffold obtained by cyclization between the C4 and C5 of the pyrimidine ring was important for ATR inhibition.

## 5. Comparative Summary of Pyridopyrimidine and Pyridopyrimidinone-Based Anticancer Agents

Several derivatives demonstrated potent inhibitory activity. Compound **1b** showed potent PIM1 kinase inhibitory activity of 0.99 µM. In compound **1b**, the 4-chloro group on the phenyl ring is essential for PIM1 inhibition. Also, the incorporation of hydrophobic centers increased the cytotoxic potential. In another study by Han et al., compound **2a** showed better PIM1 inhibitory activity of 43 nM. The piperazine ring is essential for anticancer activity in compound **2a**. Also, the presence of bulky substituents decreased the activity. Compound **5a** showed potent inhibitory activity against PI3Kα with IC_50_ of 0.183 µg/mL. In compound **5a**, the 4-chloro substitution on the phenyl ring is important for activity. The replacement of 4-chloro group with a 4-methyl group reduced the activity. Fused pyridopyrimidopyrimidines were synthesized by El-Essawy et al., and compound **14** was found to be a potent EGFR inhibitor against the EGFR^L858R/T790M^ mutant, demonstrating an IC_50_ of 60 nM. It also showed the strongest apoptotic response of 32% (total apoptotic fraction) which was higher than that of the vehicle control (0.6%). Table 2 displays the comparative summary of pyridopyrimidine and pyridopyrimidinone-based anticancer agents.

## 6. Translational Challenges and Future Perspectives

Although many pyridopyrimidine and pyridopyrimidinone derivatives have demonstrated promising anticancer activity in preclinical studies, several issues still need to be addressed before clinical use. One of the major challenges is kinase selectivity. While compounds such as **6** exhibited good selectivity toward PI3Kδ and **15a**, **15b**, and **16a** effectively targeted both wild-type EGFR and the T790M mutant, most of the reported derivatives were evaluated only against their intended kinases. As a result, their selectivity profiles and potential off-target effects remain largely unknown. Another important limitation is the limited investigation of drug resistance. Most resistance-related studies have focused only on EGFR mutations, particularly T790M, whereas compounds developed against PIM, ATR and CDKs have rarely been assessed against clinically relevant resistance mechanisms. This leaves an important research gap in understanding how these synthesized compounds might perform during long-term treatment. The lack of in vivo validation for many promising candidates is another concern. For example, compound **2a** demonstrated significant antitumor activity in a MOLM-13 xenograft model, supporting its therapeutic potential. Several potent compounds, including **5a**, were evaluated only through enzyme-based and cellular assays, making it difficult to predict whether their activity can be translated to animal models or clinical settings. In addition, information on pharmacokinetic properties, including aqueous solubility, metabolic stability, oral bioavailability, and systemic toxicity, is unavailable for many reported derivatives. These factors play a critical role in determining whether a potent kinase inhibitor can progress into drug development. Finally, despite the large number of pyridopyrimidine-based compounds reported in recent years, only a few agents, including Palbociclib and trametinib, have received FDA approval, while voxtalisib, pamapimod, and piritrexim have advanced to clinical evaluation [78]. The majority of reported compounds remain at the preclinical stage, emphasizing the need for comprehensive selectivity profiling, pharmacokinetic optimization, toxicity assessment, and well-designed in vivo studies to facilitate successful clinical translation.

## 7. Conclusions

Pyridopyrimidine has been found to be a promising heterocyclic scaffold in the development of kinase inhibitors due to its structural resemblance to the adenine moiety of ATP. The present review highlights recent advancements (2021–2026) in the development of pyridopyrimidine derivatives targeting key oncogenic kinases including EGFR, PI3K, CDK4/6, PIM, and ATR. The reported studies demonstrated that the biological activity of pyridopyrimidinone and pyridopyrimidine-based derivatives is governed not only by the core scaffold but also by the nature and position of peripheral substituents.

The clinical success of the CDK4/6 inhibitor Palbociclib further validates the therapeutic potential of the pyridopyrimidine scaffold. Reported compounds demonstrated strong antiproliferative effects, induced apoptosis, and caused cell cycle arrest across a number of cancer cell lines. SAR studies showed that strategic substitutions on the pyridopyrimidine core significantly influence potency and selectivity. Moreover, hybridization approaches and incorporation of pharmacophores such as triazoles, thiazolidinediones, and thiazoles further enhanced biological activity. Additionally, the development of dual and multi-target inhibitors offers a promising strategy to overcome drug resistance and improve therapeutic outcomes. In conclusion, pyridopyrimidine-based kinase inhibitors continue to represent a versatile platform in anticancer drug discovery. Future research should focus on optimizing selectivity and improving in vivo activity to translate these compounds into clinically viable therapeutics.

## Figures and Tables

**Table 1:** FDA approved drugs containing pyridopyrimidine core.

Parameter	FDA Approved Drug
	Palbociclib
Chemical Structure	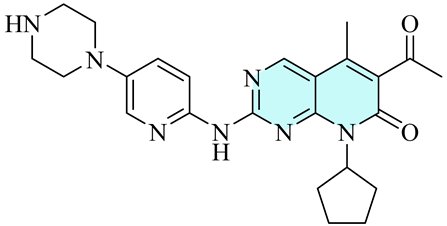
Target	CDK4/CDK6
IC_50_	11 nM (CDK4) and 16 nM (CDK6)
Selectivity	Dual CDK4/6 inhibition
Approval Year	2015
Indication	ER^+^/HER2^−^ breast cancer
Key Structural Feature	Pyridopyrimidinone core with bulky side chain
Pharmacological Note	First-in-class CDK4/6 inhibitor

**Table 2:** Comparative summary of pyridopyrimidine- and pyridopyrimidinones-based anticancer agents.

Compound	Target Kinase	Enzymatic Activity (IC_50_)	Cellular Activity (IC_50_)	Selectivity	SAR Trends	Reference
**1a**	PIM	11.4 nM	MCF-7: 0.57 µM, HepG2: 1.13 µM	PIM-1 selective	4-chloro phenyl moiety made compound most potent. Incorporation of hydrophobic centers increased the cytotoxic potential.	[49]
**1a**	PIM	17.2 nM	MCF-7: 1.31 µM, HepG2: 0.99 µM	PIM-1 selective		[49]
**2a**	PIM	PIM-1: 43 nM PIM-1: 232 nM PIM-1: 774 nM	MOLM-13: 1.83 µM	PIM-1, PIM-2, PIM-3	Piperazine ring is essential.	[50]
**5a**	PI3K	0.183, 2.227, 11.845, 0.615 µg/mL	T47D: 1.46 µM	PI3Kα PI3Kβ PI3Kγ PI3Kδ	4-chloro substitution on phenyl ring made compound most active. Unsubstituted phenyl ring showed moderate activity against T47D cell line.	[56]
**8a**, **8b**, **8c**, **8d**, **8e** and **8f**	EGFR	0.33–3.37	8a (IC_50_) MCF-7: 3.91 µM 8f (IC_50_) MCF-7: 3.30 µM	EGFR selective	SAR studies displayed that presence of EWG such as fluorine and 3,5-diCl groups, are important for inhibitory activity.	[61]
**12a**	EGFR	0.53 µM	MCF-7: 4.19 µM	EGFR selective	3, 5-dimethoxy group also displayed comparable EGFR inhibitory activity. Presence of 3, 4, 5-trimethoxy substitution made compound active.	[68]
**12b**	EGFR	0.39 µM	MCF-7: 3.52 µM	EGFR selective		
**14**	EGFR	60 nM	H1975: 0.42 µM	EGFR^L858R/T790M^	Cyano-substituted core promotes binding geometry within the EGFR pocket.	[69]
**15a**	EGFR	EGFR^wt^: 0.099 µM EGFR^T790M^: 0.123 µM	A549: 16.2 µM PC-3: 7.98 µM HCT-116: 25.61 µM	EGFR^wt^ and EGFR^T790M^	Electron-withdrawing groups like 4-chloro is essential for EGFR inhibition. Replacement with 4-methyl group slightly reduced the activity.	[70]
**15b**	EGFR	EGFR^wt^: 0.419 µM EGFR^T790M^: 0.290 µM	A549: 7.23 µM PC-3: 7.12 µM HCT-116: 70.17 µM			
**16a**	EGFR	EGFR^wt^: 0.594 µM EGFR^T790M^: 0.571 µM	PC-3: 9.26 µM	EGFR^wt^ and EGFR^T790M^	Presence of methyl group made compound less active against EGFR. Di substitution is more preferred.	[71]
**31**	ATR	15.4 nM	LoVo: 165 µM	ATR-selective	3-methylmorpholine group is essential for activity.	[77]

## Data Availability

No new data were created or analyzed in this study. Data sharing is not applicable to this article.

## Abbreviations

AKT	Protein Kinase B
ATR	Ataxia Telangiectasia and Rad3-related protein
CDK	Cyclin-Dependent Kinase
CDK4/6	Cyclin-Dependent Kinase 4 and 6
EGFR	Epidermal Growth Factor Receptor
FDA	Food and Drug Administration
IC_50_	Half Maximal Inhibitory Concentration
mTOR	Mammalian Target of Rapamycin
PI3K	Phosphoinositide 3-Kinase
PIM	Proviral Integration site for Moloney murine leukemia virus kinase
SAR	Structure–Activity Relationship
